# Lactate is useful for the efficient replication of porcine epidemic diarrhea virus in cell culture

**DOI:** 10.3389/fvets.2023.1116695

**Published:** 2023-02-13

**Authors:** Nile Wuri, Hongchao Gou, Bin Zhang, Menglu Wang, Songqi Wang, Weixiao Zhang, Haiyan He, Xuelei Fan, Chunhong Zhang, Zhicheng Liu, Letu Geri, Haiyan Shen, Jianfeng Zhang

**Affiliations:** ^1^Key Laboratory of Livestock Disease Prevention of Guangdong Province, Scientific Observation and Experiment Station of Veterinary Drugs and Diagnostic Techniques of Guangdong Province, Ministry of Agriculture and Rural Affairs, Institute of Animal Health, Guangdong Academy of Agricultural Sciences, Guangzhou, China; ^2^College of Veterinary Medicine, Inner Mongolia Agricultural University, Hohhot, China; ^3^Maoming Branch Center of Guangdong Laboratory for Lingnan Modern Agricultural Science and Technology, Maoming, China; ^4^College of Veterinary Medicine, South China Agricultural University, Guangzhou, China

**Keywords:** porcine epidemic diarrhea virus, metabolites, glucose, glutamine, lactate

## Abstract

Porcine epidemic diarrhea virus (PEDV) is a deadly pathogen infecting pig herds, and has caused significant economic losses around the world. Vaccination remains the most effective way of keeping the PEDV epidemic under control. Previous studies have shown that the host metabolism has a significant impact on viral replication. In this study, we have demonstrated that two substrates of metabolic pathway, glucose and glutamine, play a key role in PEDV replication. Interestingly, the boosting effect of these compounds toward viral replication appeared to be dose-independent. Furthermore, we found that lactate, which is a downstream metabolite, promotes PEDV replication, even when added in excess to the cell culture medium. Moreover, the role of lactate in promoting PEDV was independent of the genotype of PEDV and the multiplicity of infection (MOI). Our findings suggest that lactate is a promising candidate for use as a cell culture additive for promoting PEDV replication. It could improve the efficiency of vaccine production and provide the basis for designing novel antiviral strategies.

## 1. Introduction

Porcine epidemic diarrhea (PED), caused by the porcine epidemic diarrhea virus (PEDV), has caused huge losses to the global pig industry. This virus mainly affects neonatal piglets and its symptoms include vomiting, diarrhea, dehydration, growth retardation and high mortality (1). PEDV was first isolated in Belgium in 1978 and named CV777. In 1994, an inactivated vaccine was developed by attenuating this strain on Vero cells, which played an important role in the prevention and treatment of PED (2, 3). However, in October 2010, a new variant of PEDV was discovered in China. Starting in 2013, it caused a pandemic in the United States, Canada, Mexico and other places, before spreading to countries around the world (4, 5). As a result, commercial vaccines using the classical PEDV strains proved ineffective against the circulating strains. This led to attempts to produce a new generation of inactivated vaccines based on the PEDV variants (6, 7). However, they were plagued by the problem of low viral titers and high production costs (8). Amongst the methods attempted to increase the replication efficiency of PEDV was the addition of trypsin to the cell culture medium of PEDV-infected Vero cells. Attempts were also made to trigger the overexpression of PEDV nsp16, in order to promote viral proliferation in a manner that negatively regulates innate immunity. Hence, the development of new methods to promote viral replication in the culture medium could be a useful step in vaccine development.

Previous studies have shown that viruses alter the metabolism of host cells in order to provide optimal conditions for rapid and efficient reproduction and transmission (9–12). Viruses rely on the host cell machinery for reproduction. They promote anabolism, which results in the production of the macromolecules required for virion replication and assembly. Metabolic phenotypes conferred by viral infection often reflect the metabolic changes in host cells, such as the upregulation of nutrient consumption and anabolism to support viral replication or rapid cell growth, respectively (13). Previous studies have shown that pathogens alter the host cell metabolism, particularly the host cell's central carbon metabolism, for the benefit of their own metabolic processes (14). Shytaj et al. (15) also found that the down-regulation of glycolysis significantly reduces HIV-1 virion production. In addition, Gou et al. (16) found that glutamine metabolism affects the pseudorabies viral replication. The pathogenicity and replication of the coronavirus SARS-CoV-2 has also been reported to be dependent on glycolysis and glycosylation. Glucose, in the form of adenosine triphosphate (ATP) derived from glycolysis, acts as an energy source for the viral host cells. It is also involved in glycan formation, which is essential for the synthesis of glycoproteins during glycosylation (17). Taken together, these results demonstrate the importance of glucose and glutamine metabolism during the viral infection of cells.

In this study, we have investigated the effect of metabolites on PEDV replication in Vero cells. We found that glucose, glutamine and lactate are essential for the infection of Vero cells by classical as well as mutant PEDV strains. Lactate was even found to promote the replication of PEDV. These findings may provide useful insights for improving the virus titers of PEDV vaccine.

## 2. Materials and methods

### 2.1. Cell culture

Vero cells were cultured in Dulbecco's modified Eagle's medium (DMEM; Gibco, USA) containing 5% fetal bovine serum (FBS; Biological Industries, USA), 100 μg/ml streptomycin, and 100 units/ml penicillin at 37°C in a humidified environment with 5% CO_2_.

### 2.2. Virus culture

DMEM containing 100 μg/ml streptomycin and 100 units/ml penicillin was used for culturing PEDV on Vero cells. To explore the importance of glucose and glutamine metabolism in PEDV replication, 12-well plates confluent with a monolayer of Vero cells were seeded and infected with PEDV. The effect of metabolites on PEDV infection was analyzed using normal DMEM, glucose-free DMEM, and DMEM supplemented with glucose and lactate. The effects of glutamine on PEDV were analyzed using glutamine-free DMEM and DMEM supplemented with glutamine. The PEDV GD/HZ/2016, CV777 and TB strains of PEDV available in our laboratory were used for this study.

### 2.3. Western blot

Cells were lysed using cell lysis buffer (ThermoFisher Scientific, China) containing the protease inhibitor PMSF to prevent protein degradation. After measuring the protein concentration, 5× protein buffer was added and the proteins were denatured in a 98°C water bath. The protein samples were separated on 12% SDS–PAGE Gel SuperQuick Preparation Kit (Beyotime, China), alongside the prestained color protein marker (ThermoFisher Scientific, China). The proteins on the gel were transferred to PVDF membranes (Millipore, China) using a Trans-blot Turbo (Bio-Rad, China), and then blocked with QuickBlock™ Western Blocking Buffer (Beyotime, China). Membranes were cut, based on the bands of the protein marker, immediately after blocking. The excised membranes were incubated overnight at 4°C with PEDV N antibody (Medgene Labs, Brookings, SD, USA) and glyceraldehyde-3-phosphate dehydrogenase (GAPDH; ABclonal, China). The corresponding HRP-conjugated goat anti-mouse and anti-rabbit IgG (H + L) secondary antibodies were added the next day, followed by incubation for 1 h at room temperature. After elution, imaging was performed on a Bio-Rad imager with diluted NcmECL Ultra (NCM, China).

### 2.4. Reverse transcriptase-quantitative polymerase chain reaction

After the extraction of total RNAs using TaKaRa MiniBEST Universal RNA Extraction Kit (TaKaRa, China), the expression of the PEDV N gene was specifically detected by the reverse transcriptase-quantitative polymerase chain reaction (RT-qPCR) method (primers are listed in Table 1). HiScript^®^ II one Step qRT-PCR SYBR Green Kit (Vazyme, China) was used for detection by RT-qPCR.

**Table 1:** Primer used in the RT-qPCR study.

**Gene**	**Forward primer (5^′^-3^′^)**	**Reverse primer (5^′^-3^′^)**
***PEDV N***	GCAAAGACTGAACCCACTAA T	GCCTCTGTTGTTACTTGGAG
***ACTB***	GGACTTCGAGCAGGAGATGG	AGGAAGGAGGGCTGGAAGAG

### 2.5. Median Tissue Culture Infectious Dose (TCID_50_) measurement

Serial 10-fold dilutions (10^−1^ to 10^−8^) of the virus solution were prepared and inoculated in a 96-well plate confluent with a monolayer. After incubate for 4–5 days in a 5% CO_2_ incubator at 37°C, the cells were examined for cytopathic changes. The viral titer of each viral fluid was calculated according to the Reed-Muench equation.

### 2.6. Cell Counting Kit-8

The Vero cell viability at the time of lactate addition was detected using the Cell Counting Kit-8 (CCK-8; Abcam, China). Vero cells were grown in 96-well plate for 24–36 h, followed by incubation with DMEM containing 0, 2, 5, 10, 15, 20, 30, 40, 60, and 100 mM lactate for 24 h. Ten microliter of CCK-8 reagent was added to each well, and the cells were incubated for 1 h in a cell incubator. The optical density was measured at 450 nm using a microplate reader. The % viable cells were determined as follows: $\left(\frac{ODt}{ODc}\right)\times 100\%$, where ODt and ODc are the absorbance of treated and control cells, respectively.

## 3. Results

### 3.1. Glucose and glutamine play an important role in PEDV replication

**Figure 1:**
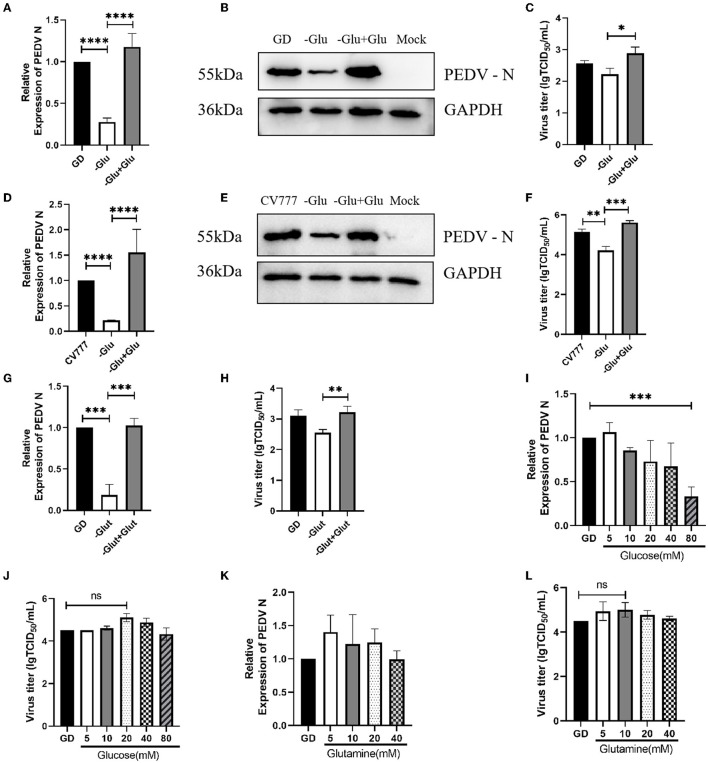
The role of glucose and glutamine in PEDV replication. **(A–C)** Glucose starvation (-Glu) inhibited the replication of PEDV GD/HZ/2016 (GD) strain in Vero cells, as seen by the analysis of the mRNA levels **(A)**, protein levels **(B)**, and viral titers **(C)** 24 h post infection. *p < 0.05; **p < 0.01; ***p < 0.001; ****p < 0.0001. **(D–F)** Glucose starvation also inhibited the replication of the PEDV CV777 strain in Vero cells, as shown by the analysis of PEDV N mRNA levels **(D)**, protein levels **(E)**, and virus titers **(F)** at 24 h post infection. **(G, H)** RT-qPCR and TCID_50_ analysis of the effect of glutamine starvation in culture medium (-Glut) and supplementation with 2 mM glutamine (–Glut+Glut) on PEDV GD/HZ/2016 replication. **(I, J)** PEDV GD/HZ/2016 N mRNA levels and viral titers after the addition of DMEM with 5, 10, 20, 40, and 80 mM glucose. **(K, L)** PEDV GD/HZ/2016 N mRNA levels and viral titers after the addition of DMEM with 5, 10, 20, and 40 mM glutamine. The meaning of ns is “no significance” *p* > 0.05.

Several previous studies have demonstrated the role of glucose in viral replication (16, 18). Since we wanted to investigate its importance in PEDV replication, we infected Vero cells, grown in DMEM medium which was deficient in or supplemented with 20 mM glucose, with the PEDV strain CV777 and the variant PEDV strain PEDV GD/HZ/2016. We observed that the glucose starvation medium produced less viral RNA than normal controls at 24 h of infection (Figures 1A, D). This result was also confirmed by western blot, which detected the expression of the PEDV-N protein (Figures 1B, E). While the virus titer was detected in the cell supernatant, it was found that the titer of the glucose-deficient group was significantly lower than that of the normal group (Figures 1C, F). The results prove that glucose plays an important role in PEDV replication. According to Hirabara et al. (19), in addition to glucose metabolism, glutamine metabolism is also altered during the reprogramming of the host cell metabolism by the virus. In order to investigate this aspect, we used glutamine-free and glutamine-supplemented DMEM to culture PEDV. It was found that the lack of glutamine also reduced the replication ability of PEDV at 24 h of infection, and the virus replication recovered after glutamine supplementation (Figures 1G, H). These results suggest that glutamine also plays an important role in PEDV replication.

Based on the above results, we speculated that normal DMEM supplemented with excess glucose and glutamine may promote PEDV replication. Subsequently, we cultured PEDV in normal DMEM with the gradient addition of glucose. However, it was found that the excessive addition of glucose did not have a significant replication-promoting effect (Figures 1I, J). Subsequent gradient addition of glutamine to normal DMEM also did not show any effect on PEDV replication (Figures 1K, L).

### 3.2. Lactate is a key factor in the glycolytic pathway to promote PEDV infection

**Figure 2:**
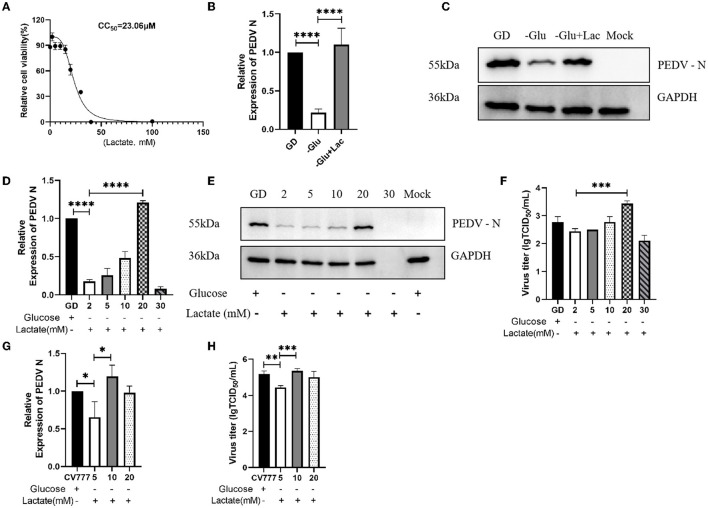
Lactate is a key compound in the glycolytic pathway for the promotion of PEDV infection. **(A)** The CC_50_ was calculated from the dose-response curves generated from the CCK-8 experimental data and subjected to nonlinear regression analysis. **(B, C)** Enhanced replication of PEDV GD/HZ/2016 (GD) strain was observed on supplementation of glucose-free DMEM with 20 mM lactate (–Glu+Lac), as shown by the analysis of the PEDV N mRNA levels **(B)** and protein levels **(C)** at 24 hpi. **(D–F)** Replication of the PEDV GD/HZ/2016 strain was restored on supplementation of glucose-free DMEM with 5, 10, 20, and 30 mM lactate, as shown by the analysis of the PEDV N mRNA levels **(D)**, protein levels **(E)**, and viral titers **(F)**. **(G, H)** The analysis of the PEDV N mRNA levels **(G)** and viral titers **(H)** showed that the supplementation of 5, 10, and 20 mM lactate in glucose-free DMEM restored the replicative capacity of the PEDV CV777 strain. The significance of ^*^p < 0.05; ^**^p < 0.01; ^***^p < 0.001; ^****^p < 0.0001.

A previous study has reported that lactate plays a role in the infection of Influenza A virus in human airway epithelial cells (20). In view of this, we attempted to investigate whether lactate plays an important role in the infection of Vero cells by PEDV. We first examined the toxicity of lactate to Vero cells using the CCK-8 assay. The 50% cell cytotoxicity (CC_50_) calculations showed that lactate was not excessively toxic to cells up to a concentration of 23.06 mM (Figure 2A). Since one molecule of glucose is broken down into two molecules of lactate through glycolysis, we supplemented glucose-free DMEM with 20 mM lactate. As expected, the PEDV titers recovered in the lactate-supplemented group, when compared to the glucose-depleted DMEM group, and surpassed the levels of the normal DMEM group (Figures 2B, C). In order to establish the concentration of lactate producing the most pronounced promoting effect, we added lactate to glucose-depleted DMEM medium in a gradient manner. The results showed that the addition of 20mM and 10 mM lactate to Vero cells infected with PEDV GD/HZ/2016 and PEDV-CV777, respectively, produced the maximum promoting effect (Figures 2D–H).

### 3.3. The promoting effect of lactate on different strains of PEDV is concentration-specific

**Figure 3:**
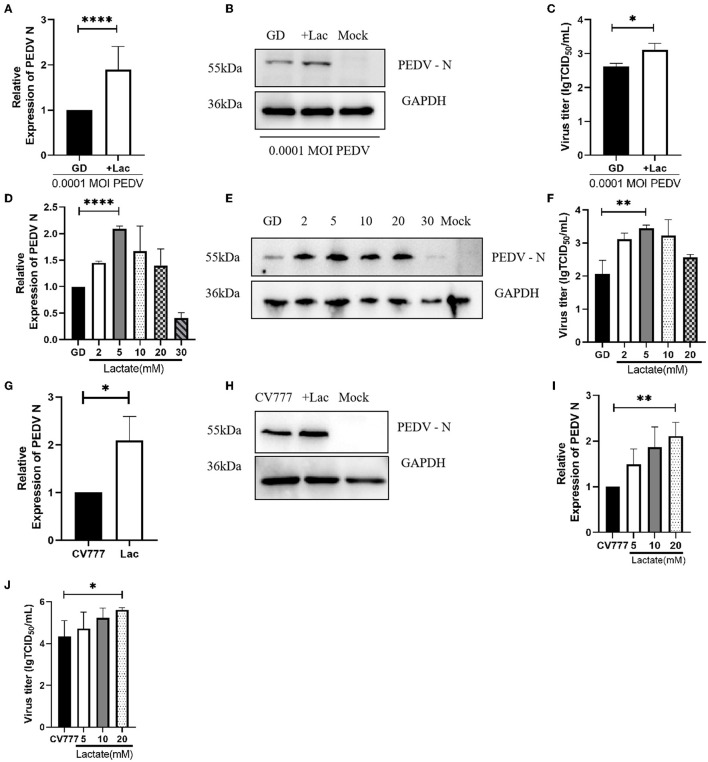
The promoting effect of lactate on different strains of PEDV is concentration-specific. Analysis of PEDV N mRNA levels **(A)**, protein levels **(B)**, and viral titers **(C)** in Vero cells grown in normal DMEM supplemented with 10 mM lactate and infected with 0.0001 MOI PEDV GD/HZ/2016 strain. **(D–F)** The effect of the supplementation of normal DMEM with 2, 5, 10, 20, and 30 mM lactate on the replication of PEDV GD/HZ/2016 strain, as shown by the analysis of the PEDV N mRNA levels **(D)**, protein levels **(E)**, and viral titers **(F)** in Vero cells. The addition of 10 mM lactate to normal DMEM promoted the replication of PEDV CV777 strain, as shown by the PEDV N mRNA **(G)** and protein levels **(H)**. **(I, J)** The increase in the PEDV CV777 replication on addition of 5, 10, and 20 mM lactate to normal DMEM was found to be concentration-dependent, as shown by the PEDV N mRNA levels **(I)** and viral titers **(J)**. ^*^p < 0.05; ^**^p < 0.01; ^****^p < 0.0001.

After the above results demonstrated the importance of lactate in PEDV replication, we attempted to evaluate the effect of excessive addition of lactate on PEDV replication. The cells were grown in normal DMEM supplemented with 10 mM lactate, and the virus was collected 24 h after infection. The results of the PEDV N mRNA assay showed that lactate promoted the replication of both PEDV GD/HZ/2016 and PEDV-CV777 strains (Figures 3A, G). The findings were also supported by the results of the western blotting (Figures 3B, H). Lactate promotes PEDV GD/HZ/2016 in the detection of viral titers (Figure 3C). The gradient addition of lactate in normal DMEM was performed in order to determine the concentration of lactate having the greatest promoting effect. The viral RNA and viral titers were detected 24 h after infection. The results showed that 10 mM of lactate had the most pronounced promoting effect on the PEDV GD/HZ/2016 strain (Figures 3D–F), while 20 mM lactate had the maximum effect on PEDV-CV777 strain (Figures 3I, J). In conclusion, the addition of 10–20 mM lactate to normal DMEM can promote PEDV replication.

### 3.4. Lactate promotes the replication of PEDV variant strains in Vero cells

**Figure 4:**
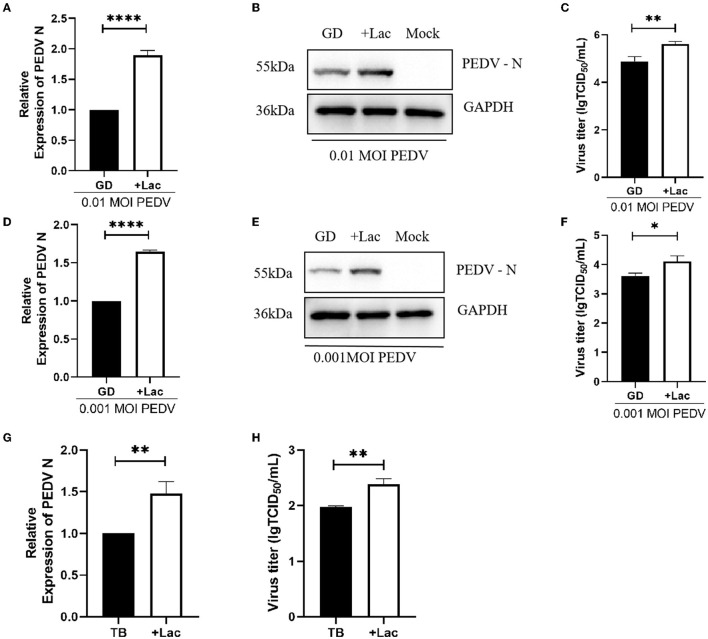
Lactate promotes the replication of PEDV variant strains in Vero cells. **(A–C)** Enhanced viral replication of 0.01 MOI PEDV GD/HZ/2016 strain in Vero cells after addition of 10 mM lactate to normal DMEM, as shown by the analysis of the PEDV N mRNA levels **(A)**, protein levels **(B)**, and viral titer **(C)**. **(D–F)** Ten millmolar lactate has a viral replication-promoting effect on the PEDV GD/HZ/2016 strain at 0.001 MOI. PEDV N mRNA levels **(G)** and viral titers **(H)** indicated that 10 mM lactate has a promoting effect on laboratory-preserved variant PEDV TB strains. ^*^p < 0.05; ^**^p < 0.01; ^****^p < 0.0001.

To confirm that lactate promotion has the same effect on PEDV GD/HZ/2016 strain with a different MOI, Vero cells were infected with PEDV GD/HZ/2016 at MOI of 0.01, 0.001, and 0.0001. A normal DMEM group and a lactate-added DMEM group were set at the same time. The results showed that lactate had a promoting effect on PEDV GD/HZ/2016 strains with different MOIs (Figures 3A–C, 4A–F).

To demonstrate the ability of lactate to promote PEDV of other clinical strains, experiments were performed on PEDV TB strains belonging to the GII genotype isolated from clinical samples. Viruses were collected 72 h after infection, and the viral RNA and titer assays showed that lactate promotes PEDV replication regardless of serotype (Figures 4G, H).

## 4. Discussion

Vaccination is an efficient and cost-effective step in the prevention and control of PED to reduce the economic losses associated with this epidemic (2, 3, 21). However, consistently high viral titers are difficult to obtain during the propagation of PEDV *in vitro*, which poses a challenge in vaccine development. Therefore, attempts are being made to elucidate the compounds which could be added to the cell culture medium for promoting virus production. This could help overcome some of the challenges in vaccine production including high production cost, low efficacy of antigens and poor immune response due to low antigen yield.

Glucose and glutamine are the main carbon sources for energy balance and biosynthesis in mammalian cells, and are the main substrates in the culture medium. Many studies have demonstrated that an increase in the uptake of glucose and glutamine during viral infection, and have highlighted their importance in virus replication (22–24). In our study, we tested the effect of glucose and glutamine on the proliferation of PEDV in Vero cells using a low-glucose and low-glutamine environment. We found that viral replication was significantly lower in the low-glucose and low-glutamine group, when compared to the normal group. The result suggests that glucose and glutamine affect PEDV replication and play an important role in PEDV infection.

In view of the above results, we speculated that the exogenous addition of glucose and glutamine would promote the replication of PEDV. However, we did not observe any promotion of PEDV replication upon the addition of excess glucose or glutamine. This is consistent with previous findings with respect to the foot-and-mouth virus, which showed that virus production was unaffected by the presence of excess glucose and glutamine (25). However, Guo et al. (26) indicated that the exogenous addition of glucose promoted the production of Singapore grouper iridovirus (SGIV) viroids, while the exogenous supplementation with glutamine had no effect. High glucose environments were reported to promote viral replication in enteroviruses and Usutu virus (USUV) (27, 28). Taken together, although glucose and glutamine have been shown to be essential substrates in PEDV replication, they do not promote PEDV replication when added in excess.

Lactate is produced not only in the metabolism of glucose but also by that of glutamine. The fermentation of glutamine to lactate is called the Warburg effect (29). A previous study had reported that lactate plays a major role in the replication of Vesicular Stomatitis Virus (VSV), and it can promote the replication of Influenza A (IAV) (20, 30). In our study, we supplemented lactate in glucose-free DMEM. It was found to have a restorative effect on PEDV replication, thus highlighting its importance in the process. The importance of lactate has also been reported in studies on other viruses (20, 30–32). In addition, we added lactate to the normal medium of genotype I and II PEDV-infected Vero cells and found that lactate has the effect of promoting viral titers. This finding suggests that lactate is a promising substrate for improving PEDV titers during vaccine production. Our experiments demonstrated that lactate has a promoting effect on both genotype I and II PEDV strains. We also added lactate when infecting Vero cells with PEDV GD/HZ/2016 strains of different MOIs. The results showed that the promotion of PEDV by lactate was independent of the initial inoculation dose. Furthermore, the replication of clinical PEDV TB strains (genotype II) verified this phenomenon. Taken together, the results suggest that lactate has a positive effect on PEDV replication.

In this study, we have used lactate, a metabolite produced during the virus-host interaction, for the first time to promote PEDV replication and increase viral titers. Glucose and glutamine in the medium played an important role during the PEDV infection of Vero cells. Interestingly, glucose and glutamine did not enhance PEDV replication when added in excess. The addition of lactate in excess promoted viral replication, which could be advantageous in the production of vaccines.

## Data availability statement

The original contributions presented in the study are included in the article/supplementary material, further inquiries can be directed to the corresponding authors.

## Author contributions

NW performed the data analysis and drafted the manuscript. NW, HG, BZ, and MW conducted the experiments. HG, CZ, ZL, and LG participated in the data analysis. JZ and HS conceptualized this study. SW, WZ, HH, and XF prepared the materials for the experiments. All authors contributed to the article and approved the submitted version.
